# Unlocking New Potential in Tea Pest Management Using Novel Paraffinic Hydrocarbon-Based Tank-Mix Adjuvant by Boosting Insecticide and Acaricide Efficacy

**DOI:** 10.3390/insects17090882

**Published:** 2026-08-24

**Authors:** Somnath Roy, Debashis Roy, V. Rakesh, Sourajit Bayen, Sankhadeep Mondal, Sarvesh Singh Tomar, Ashish Kumar Mishra, Venkatesan Selvaraj, Abhay Kumar Pandey

**Affiliations:** 1 Tea Research Association, Tocklai Tea Research Institute, Jorhat 785008, Assam, India; somnathento@gmail.com (S.R.); rksh1998@gmail.com (V.R.); picklu92@gmail.com (S.B.); sankhadeep810@gmail.com (S.M.); director@tocklai.net (V.S.); 2 R&D Center, Bharat Petroleum Corporation Limited, Sewree, Mumbai 400015, Maharashtra, India; tomarss@bharatpetroleum.in (S.S.T.); ashishkmishra@bharatpetroleum.in (A.K.M.); 3 Tea Research Association, North Bengal Regional R&D Center, Nagrakata 735225, West Bengal, India

**Keywords:** adjuvant, bioassay, agrochemicals, *Hyposidra talaca*, *Oligonychus coffeae*, tea, toxicity, SEM, yield

## Abstract

This study investigates the effect of a brand-new tank-mix adjuvant, Spray Adjuvant—Paraffinic Hydrocarbon Surfactant Complex (SA-PHSC), in enhancing the efficacy of commonly used insecticides and acaricides against two significant pests of tea plantations, the black looper (*Hyposidra talaca*) and the red spider mite (*Oligonychus coffeae*). Given the pest’s major influence on tea cultivation, which can result in output reductions of up to 55%, this study addresses mounting concerns of pesticide resistance and climate change. Laboratory bioassays showed that the combination of SA-PHSC with the insecticides quinalphos, deltamethrin, and emamectin benzoate, and acaricides etoxazole and fenpyroximate, significantly increased the mortality rates of *H. talaca* larvae (76.67–100.0%) and *O. coffeae* adults (100.0%). Additionally, enhancements were noted in the mechanisms of pest cuticular penetration and contact toxicity. These observations were corroborated by field trials, which demonstrated enhanced control of *H. talaca* (70.47–97.63%) and *O. coffeae* (75.87–96.59% reduction) populations, as well as increased green tea leaf yields, as a result of SA-PHSC treatment in conjunction with the evaluated pesticides. In addition, the study evaluated the phytotoxicity and organoleptic attributes of the made tea after application, confirming the safety and absence of any off-flavors in the final product (scoring 6.6–7.0). These findings indicate that SA-PHSC could serve as a valuable adjuvant for sustainable pest management in tea cultivation, as it has the ability to optimize the application of currently recommended insecticides and acaricides and restore pest susceptibility.

## Simple Summary

This study evaluates the efficacy of Spray Adjuvant—Paraffinic Hydrocarbon Surfactant Complex (SA-PHSC) as a tank-mix adjuvant. Its primary objective is to improve the efficacy of insecticides and acaricides against the black looper, *Hyposidra talaca*, and the tea red spider mite, *Oligonychus coffeae*, in tea plantations, where these pests are responsible for substantial yield reductions. The combination of SA-PHSC with specific pesticides led to enhanced pest control strategies and elevated mortality rates, as evidenced by laboratory bioassays. Field trials corroborated these observations, demonstrating substantial reductions in pest populations and increased yields of green tea leaves. Additionally, the product’s safety was verified through assessment of its organoleptic characteristics and phytotoxicity, which implies that SA-PHSC has the potential to be a valuable tool for the implementation of more sustainable arthropod pest management practices in tea agro-ecosystems.

## 1. Introduction

Tea, *Camellia sinensis* (L.) O. Kuntze, is a competently managed perennial monoculture crop cultivated on both large- and small-scale plantations. It is grown on over 2.71 million ha in more than 34 countries across Asia, Africa, Latin America, and Oceania to produce 3.22 million metric tons of made tea annually [1]. An average yield loss of about 5% to 55% is mainly caused by arthropod pests [2,3]; however, up to 100% crop loss can also occur in severe cases [4]. Global climate change is causing an outbreak of new pests, resulting in rapid changes in pest dynamics, and thereby new challenges are being faced by tea growers [3]. Among the major pests, the black looper, *Hyposidra talaca* Walker (Lepidoptera: Geometridae), and the red spider mite (RSM), *Oligonychus coffeae* Nietner (Acari: Tetranychidae), are known for their damage to North-East Indian tea plantations, including the Darjeeling foothills of eastern Himalayan Terai-Dooars and Assam, causing huge crop losses [3,5]. Increased infestation of these pests in recent years might have occurred due to repeated use of synthetic pesticides and subsequent development of resistance [1,6,7], making them the most dominant species in the tea ecosystem [8].

*H. talaca* is a threat to tea plantations due to its rapid development and multiplication (8 generations year^−1^), short life cycle, ability to multiply and develop during winters, and lack of effective natural enemies [5,9]. The caterpillars can adapt to different alternative host plants due to their polyphagous nature [1]. In tea, the larvae feed on the young harvestable leaves, causing yield loss and destroying the photosynthetic potential of the mature leaves [10]. Additionally, *O. coffeae* is the most damaging mite pest, causing reddish-brown coloration on upper surface of mature leaves. Although RSM infestation is observed in tea plantations throughout the year, their number increases during the hot summer months and gradually declines during the rainy season, and under optimal environmental conditions there may be up to 22 overlapping generations [11]. Adults, nymphs and larvae feed on the sap of young leaves, lacerate leaf tissues, hence turning the leaves reddish-bronze in color. Infestation by RSM decreases the photosynthetic activity of leaves thereby causing them to wilt completely.

Overreliance on agrochemicals and injudicious application of synthetic pesticides for pest control by the tea planters, however, is a burden to both planters and the environment, resulting in primary and secondary pest outbreaks, the evolution of insecticide resistance, environmental contamination, and residue problems in consumable made tea [3]. However, the application process (spraying) may lead to pesticide droplet evaporation, drifting, and splashing, which decrease binding to leaves and pollute the surrounding environment, including non-target organisms, natural enemies, and pollinators [12]. Therefore, methods to increase pesticide efficacy while minimizing environmental loss through target specificity have become a major research focus [13]. Recently, the use of tank-mix adjuvants in pesticide spray mixtures, often known as ‘Surface Active Agent’, has been identified as an effective strategy to improve pesticide effectiveness and minimize environmental risks [14]. Besides improving the pesticide effectiveness in the plant system, several types of tank-mix adjuvants, such as surfactants, organosilicon, mineral oils, and plant essential oils, are commonly used in the pest and disease management of crops to enhance the pesticide activity in insect or fungus body [15]. The Spray Adjuvant—Paraffinic Hydrocarbon Surfactant Complex (SA-PHSC), developed based on highly refined paraffinic hydrocarbon with additives, is an oil adjuvant or a crop oil concentrate that can be used in agriculture to enhance the effectiveness of pesticides and other active ingredients. The SA-PHSC is chemically an ethoxylated non-ionic surfactant complex with a hydrocarbon-based formulation for horticultural use and is a joint patented product by Bharat Petroleum Corporation Limited and Tea Research Association (Indian Patent No. 521345; International Publication No. WO 2024/228046 A1). While a number of studies indicate fairly well the impact of adjuvants on boundary phenomena for herbicides and fungicides, only a few studies are available for insecticides [16]. One of the reasons for the shortage is that increasing the activity of insecticides with adjuvants is much more complex than improving the efficacy of other compounds such as herbicides [16]. Previous reports suggested that mixing of adjuvants with organophosphate insecticides can enhance the mortality of some insect pests and fungal pathogens through synergistic interactions [17,18]. However, because of the complex physicochemical interactions between different insecticides with different adjuvants, general principles and assumptions provide a starting point only, while real performance optimization is reached on a ‘case by case’ basis [16]. Quinalphos, deltamethrin and emamectin benzoate, an organophosphate, pyrethroid, and avermectin compound, respectively, are characterized by CIB- and RC-approved registered insecticides for tea insect pest management under Plant Protection Code (PPC) ver. 18.0, Government of India [19]. Similarly, etoxazole and fenpyroximate are two compounds with acaricidal properties and registered to control different phytophagous mites. These formulated products are also known to be effective against the looper and mite complex in tea ecosystem [20]. However, several reports addressed the incidence of poor susceptibility of *H. talaca* and *O. coffeae* to the respective insecticides and acaricides from several tea plantations of the North-Eastern Himalayan foothills of India in the last few years, which is a major concern for sustainable tea pest management. Nevertheless, no information is available on the effect of the application of these molecules in combination with tank-mix adjuvants in plant or insect system as a strategy to boost up the field effectiveness of existing insecticides, neither on bio-efficacy nor on their potential mechanisms of action and phytotoxicity.

The present study aimed to investigate the contribution of tank-mix SA-PHSC on laboratory toxicity and field efficacy of quinalphos, deltamethrin and emamectin benzoate against *H. talaca* and of etoxazole and fenpyroxymate against *O. coffeae*. The mobility of tank-mix SA-PHSC with the tested insecticides and acaricides through cuticular penetration of tea looper caterpillar and RSM adult, respectively, was studied using scanned electron microscopy (SEM). Besides, the phytotoxicity and taint effect on made tea were also determined for the SA-PHSC when applied with the formulated compounds for the adaptation at the farmers’ level.

## 2. Materials and Methods

### 2.1. Preparation of Spray Adjuvant—Paraffinic Hydrocarbon Surfactant Complex (SA-PHSC)

#### 2.1.1. Formulation

The SA-PHSC is a paraffinic hydrocarbon-based formulation developed through advanced petroleum refining and precision surfactant engineering to function as a synergistic adjuvant for agrochemical applications. Its formulation integrates ultra-refined paraffinic hydrocarbons with a proprietary non-ionic ethoxylated surfactant system, thereby combining superior physicochemical stability with enhanced pesticide delivery efficacy.

#### 2.1.2. Environmental and Safety Attributes

The ultra-refined paraffinic hydrocarbons employed in SA-PHSC belong to the mineral oil saturated hydrocarbon (MOSH) class, devoid of carcinogenic mineral oil aromatic hydrocarbons (MOAH). Rigorous residue analysis (GC-FID, GCMS) confirmed the absence of detectable hydrocarbon residues in made tea post-application. Collectively, the molecular refinement, surface-active engineering, and environmental safety profile of SA-PHSC position it as a next-generation adjuvant for sustainable integrated pest management in tea agro-ecosystems. Refining and production process, and the integration and adjuvant mechanism of SA-PHSC is provided in File S1.

### 2.2. Insecticides and Acaricides

Commercial formulations of quinalphos 25% EC (Ekalux; Syngenta, Pune, India), deltamethrin 2.8% EC (Decis; Bayer Crop Science Ltd., Thane, India), and emamectin benzoate 5% SG (Proclaim; Syngenta, Pune, India) were taken as the insecticidal treatments. Additionally, etoxazole 10% SC (Borneo; Sumitomo Chemical India Ltd., Mumbai, India), and fenpyroxymate 5% EC (Formite; Parijat Industries, New Delhi, India) were used as acaricide molecules. All the formulated products were procured from a licensed pesticide dealer and used in the present study for laboratory and field experiments. The detailed treatment schedules for both *H. talaca* and *O. coffeae* are shown in Table 1. In tea, there are only two compounds, sesame oil (0.50%) and soapnut (0.05%), are recommended and approved by the PPC as adjuvant or surfactant [19]. The present study was undertaken with this newly developed adjuvant compound, SA-PHSC, due to the continuous poor performances of the existing and registered commercial adjuvants in pesticide efficacy in the field, and the recommended adjuvants were not included in the treatment schedules.

### 2.3. Collection and Maintenance of Insects and Mites

The 2nd and 3rd instar larvae (average length 3.0 ± 1 cm) of the black looper, *H. talaca* were taken for the laboratory bioassay in the current study. Larvae were collected manually from the untreated bushes of Kharikatia Tea Estate, Jorhat, Assam (26.6299° N, 94.2667° E) and were reared on fresh and pesticide-free tender tea foliage in the entomology laboratory of Tocklai Tea Research Institute, Jorhat, Assam [21]. Besides, the presence of reddish bronze leaves confirms the attack of RSM, *O. coffeae*, in the tea plantation and was selected for field sampling [11]. Infested leaves with RSM populations were collected from the untreated bushes of Borbhetta Tea Estate, Jorhat, Assam (26.7179° N, 94.2030° E) and cultured on fresh and untreated tea leaves in the entomology laboratory of Tocklai Tea Research Institute, Jorhat, Assam. Both the insects and mites were maintained under the controlled laboratory conditions of 25 ± 5 °C temperature, 70–80% RH, and a photoperiod of 14:10 (L:D).

### 2.4. Laboratory Bioassay

Laboratory bioassays of quinalphos, deltamethrin, and emamectin benzoate alone in the recommended doses and in combination with SA-PHSC at 0.25% concentration (2.5 mL L^−1^ of water) against *H. talaca* were conducted using the standard ‘leaf-dip’ method [22]. Shoots with semi-matured leaves were dipped in each treatment solution for 10 s and were air-dried at room temperature for 10 min. Their base was wrapped with wet absorbent cotton and kept in glass vials containing tap water, and the setup was placed in a Petri dish lined with blotting paper. Ten *H. talaca* larvae of 3rd and 4th instars were taken from the collected population culture and were released on the treated leaves as a replicate using a camel-hair brush. Then, the assembly was covered with a glass chimney roofed with a muslin cloth. There were seven treatments, including the control (tap water, pH 7.0) and each was replicated three times (Table 1). Post-treatment observations were taken after 24 and 48 h.

The laboratory toxicity evaluation of etoxazole and fenpyroximate alone in the recommended doses and in combination with SA-PHSC at 0.25% concentration (2.5 mL L^−1^ of water) against *O. coffeae* was conducted by following the methods of acaricidal and ovicidal tests described by Roy et.al [23]. Fresh tea leaves were cut into circular pieces (2 cm diameter) and dipped in the treatment solutions for 10 s. Then, the treated leaves were air-dried at room temperature for 10 min and placed on moist absorbent cotton in each Petri plate. Ten RSM adults were introduced on the treated leaf disc in each Petri plate and sealed with a muslin cloth. Five different treatments were set, including the control (tap water, pH 7.0) and each was replicated four times (Table 1). The mortality data of RSM were recorded after 24 and 48 h exposure to the tested treatments. Both laboratory bioassay experiments of *H. talaca* and *O. coffeae* were conducted at 25 ± 5 °C temperature, 70–80% RH and a photoperiod of 14:10 (L:D).

The concentration of SA-PHSC used in the laboratory experiments (0.25%) in combination with chemicals, were determined based on the mortality data of SA-PHSC alone for both the *H. talaca* and *O. coffeae*. Basically, the concentration of SA-PHSC was adjusted as, the maximum concentration at which no significant mortality of the target pests was observed in the laboratory conditions, when the SA-PHSC was used alone. Therefore, no intrinsic toxic or sublethal effects of SA-PHSC alone were there in the laboratory conditions at 0.25%, when used in combination with pesticides. The compound only enhanced the efficacy of the tested chemical insecticides or acaricides through increased penetrations of the molecule both in plant as well as in insect and mite systems.

### 2.5. Field Trial

Field evaluation of the tested insecticides and acaricides alone and in combination with SA-PHSC at 0.50% (5 mL L^−1^ of water) concentration against *H. talaca* and *O. coffeae*, respectively, was conducted at Soraipani Tea Estate, Assam (26.5456° N, 94.2276° E) and Bengdubi Tea Estate, West Bengal (26.7277° N, 88.2995° E) over two spring-summer seasons (March–May) of 2024 and 2025 (Table 1). In the field conditions, both the *H. talaca* and *O. coffeae* can infest the same plantation simultaneously, and individual application for each independent target pest is practically not possible. So, the dose of the SA-PHSC was fixed at a point (0.50%), which can be utilized in the field by mixing with pesticides without having any independent toxic effect against any of the target pest. Additionally, the reason behind using the higher dose of SA-PHSC than the lab conditions basically due to the abiotic and biotic environmental factors responsible for the spray efficacy in the open field conditions, rather than the controlled laboratory conditions. In both locations, pruned tea bushes were selected for field bio-efficacy experiments with no record of pesticide application since the last pruning. Experiments were laid in Randomized Block Design (RBD) independently for each target pest with three replications for *H. talaca* and four replications for *O. coffeae*, each having a plot size of 5 m × 4 m, comprising a minimum of 50–60 bushes per replication [24]. Each randomized block was demarcated and marked using reflecting ribbon at both the experimental sites, and the total spray volume required for each treatment was calculated based on the recommended rate of 400 L water (pH 7.0) per ha [24]. Spraying was done using a hand-operated knapsack sprayer fitted with hollow cone NMD 60,450 nozzles (droplet diameter 1.6 mm, droplet size 140 µ, discharge rate 450 mL min^−1^, pressure 40 psi) [25]. For *H. talaca*, data were recorded by counting the larval population from twenty randomly selected and tagged bushes in each replication 24 h before (pre-treatment count) and 3 and 7 days after the spraying. However, twenty-five randomly selected semi-matured leaves (4th from the top) from twenty tagged bushes in each replication were selected for *O. coffeae*, and the observations on mite incidence (motile individuals) were taken 24 h before (pre-treatment count), and 7 and 10 days after the imposition of different treatments using a hand lens [26]. The post-application percent reduction/increase in looper or RSM population was enumerated following the formula given by Henderson and Tilton [27]:

(1) $$Reduction\left(\%\right)=\left\{1-\frac{\left(T_{a}\times C_{b}\right)}{\left(T_{b}\times C_{a}\right)}\right\}\times 100$$

where *T_a_*: mite population in treated leaf after treatment; *T_b_*: mite population in treated leaf before treatment; *C_a_*: mite population in control leaf after treatment; *C_b_*: mite population in control leaf before treatment.

Yield of green tea leaves in each replicated plot was recorded separately for *H. talaca* and *O. coffeae* during the first (14 days) and second (21 days) round of plucking after the application of different treatments at both the experimental sites in two years.

### 2.6. Scanning Electron Microscope (SEM) Imaging

For SEM imaging, the third instar larvae of *H. talaca* and adults of *O. coffeae* were treated with 0.25% concentration of SA-PHSC and neutral tap water (control), and the images were captured using a high-performance field emission scanning electron microscope (Zeiss Gemini SEM Sigma 300VP FE-SEM; SR/PURSE Phase 2/34, Carl Zeiss, Oberkochen, Germany). Treatment was undertaken independently by spraying the individuals at the aforementioned dose, and the insect and mite samples were collected after 24 h for microscopy. Both the treated and untreated (control) samples were collected and immersed in 2.5% of glutaraldehyde solution for 24 h for fixation. Then, the specimens were immersed in a series of alcohol gradients (30%, 50%, 70%, 90% and 100%) for dehydration with each step lasting for about 15 min, and the final dehydration was done by immersing the samples in isoamyl alcohol [28,29]. To improve the scanning electron microscopy (SEM) findings, Critical Point Drying (CPD) was employed to eliminate moisture from the looper and red spider mite tissue. The specimen was thoroughly dissected and placed on SEM stubs, which were subsequently coated with gold using a coater to prevent development of charge on the specimen surface. SEM analysis uses a focused electron beam with an energy range of 0.2–40 keV to acquire detailed and highly magnified images of the surface topography of *H. talaca* larvae and *O. coffeae* adults.

### 2.7. Phytotoxicity, Organoleptic Evaluation and Residue Analysis of Made Tea

Phytotoxicity of SA-PHSC at 1%, 2%, and 4% concentrations was evaluated separately during the field trials on young, semi-mature, and mature tea leaves, and observations were taken for epinasty, hyponasty, leaf-tip injury, leaf-surface injury, wilting, vein clearing, etc., on a 0–10 scale up to 63 days of spraying as per the established guidelines [23]. To determine the taint effect of SA-PHSC on black tea, organoleptic evaluation of made tea was performed using three different concentrations (0.5%, 1.0%, and 2.0%) and a control (0.0%). A hand-operated knapsack sprayer with the aforementioned characteristics was used for spraying different concentrations of SA-PHSC. On the 7th and 14th days post-spraying, treated tea shoots were collected and processed independently in a mini-CTC machine. The samples were sent to tea testers for the organoleptic test to determine whether the taint was positive or negative by considering leaf infusions and liquor strength. Three independent blind tests were performed using a 10-scale score. A score of 1–2 was regarded as poor, 3–5 was considered moderate, 6–8 was considered good, and 10 was considered very good [30].

### 2.8. Data Analysis

Laboratory and field bio-efficacy data of different treatment schedules against *H. talaca* and *O. coffeae* and yield of green tea leaves were subjected to statistical analysis with ANOVA using SPSS software (version 18.0: SPSS Inc., Chicago, IL, USA). Data that lacked normality were transformed using arcsine or square root transformation before subjected to statistical analysis. Mean values were separated by Duncan’s Multiple Range Test (DMRT) at *p* < 0.05 for interpretation of the results [31].

## 3. Results

### 3.1. Laboratory Toxicity of Different Treatments Against H. talaca and O. coffeae

**Figure 1:**
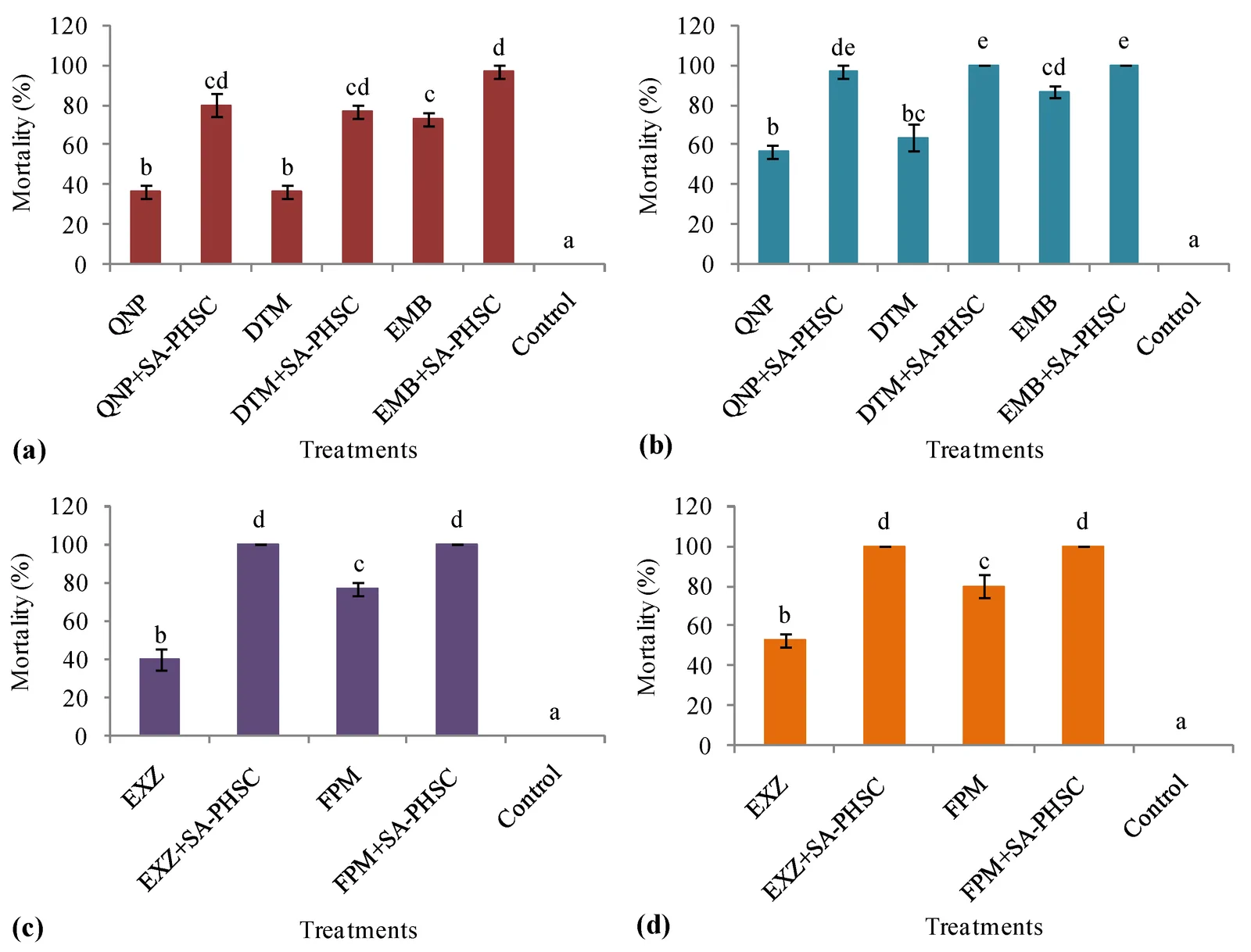
Relative toxicity of different insecticides/acaricides alone and in combination with SA-PHSC (0.25%) on *H. talaca* larvae at (**a**) 24 h; (**b**) 48 h bioassay (three replications); and *O. coffeae* adults at (**c**) 24 h; (**d**) 48 h bioassay (four replications) under laboratory conditions. Data are shown as mean ± SE. Different letters in a single parameter indicate significant differences (*p* < 0.05). Refer to Table 1 for the details of treatments.

**Figure 2:**
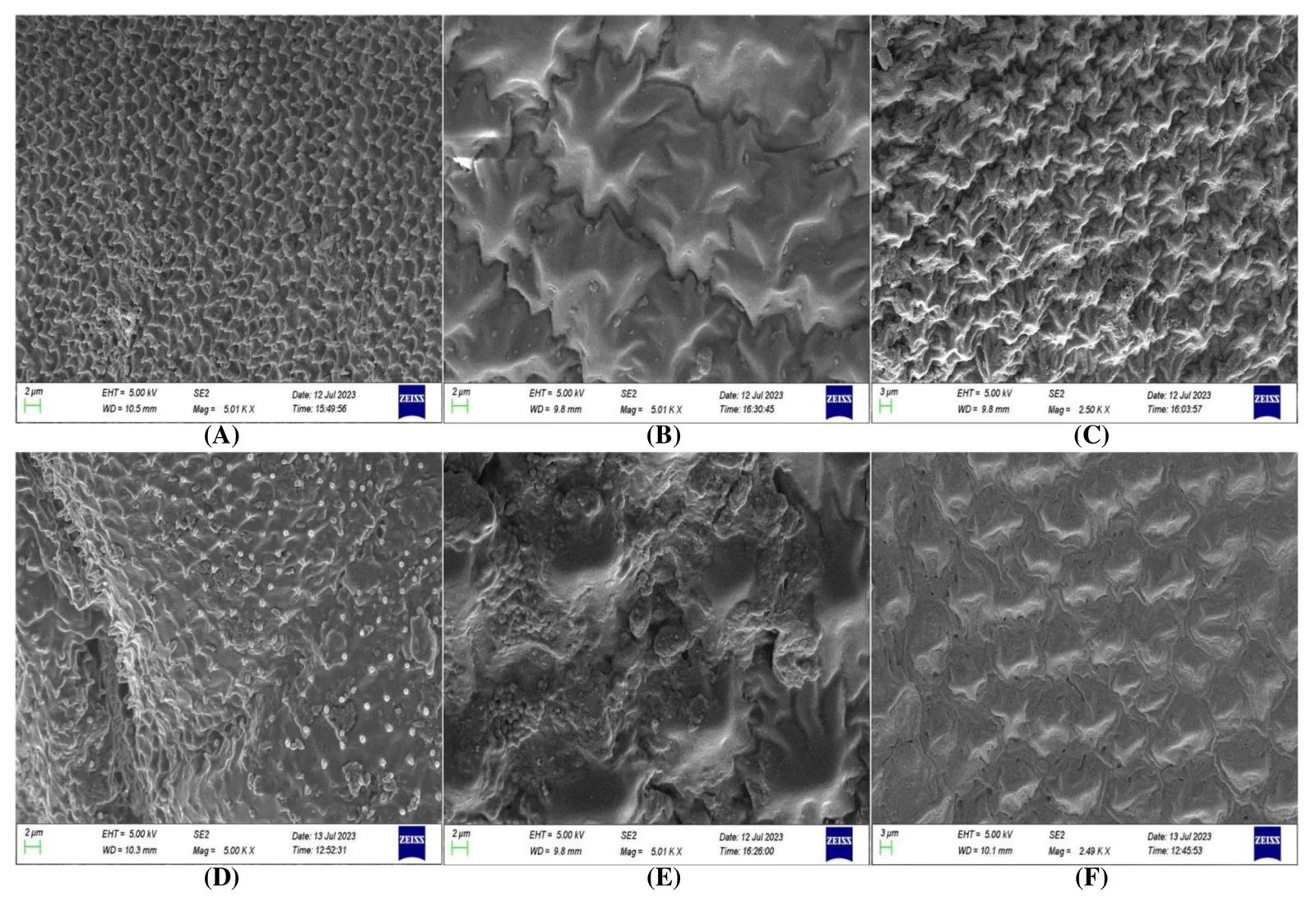
Scanning Electron Microscopy (SEM) photographs of larval cuticle of *H. talaca* with and without SA-PHSC 0.25% treatment. Section (**A**,**B**) indicate no abnormality for the control treatment at 5K× magnification; (**C**) normal cuticle before SA-PHSC treatment at 2.5K× magnification; (**D**) post-treatment larval cuticle with numerous open micro pores on the cuticular surface; (**E**) rupture and decaying of cuticular surface; (**F**) morphological deformities throughout the larval skin surface with loose cuticular binding and poor compactness.

**Figure 3:**
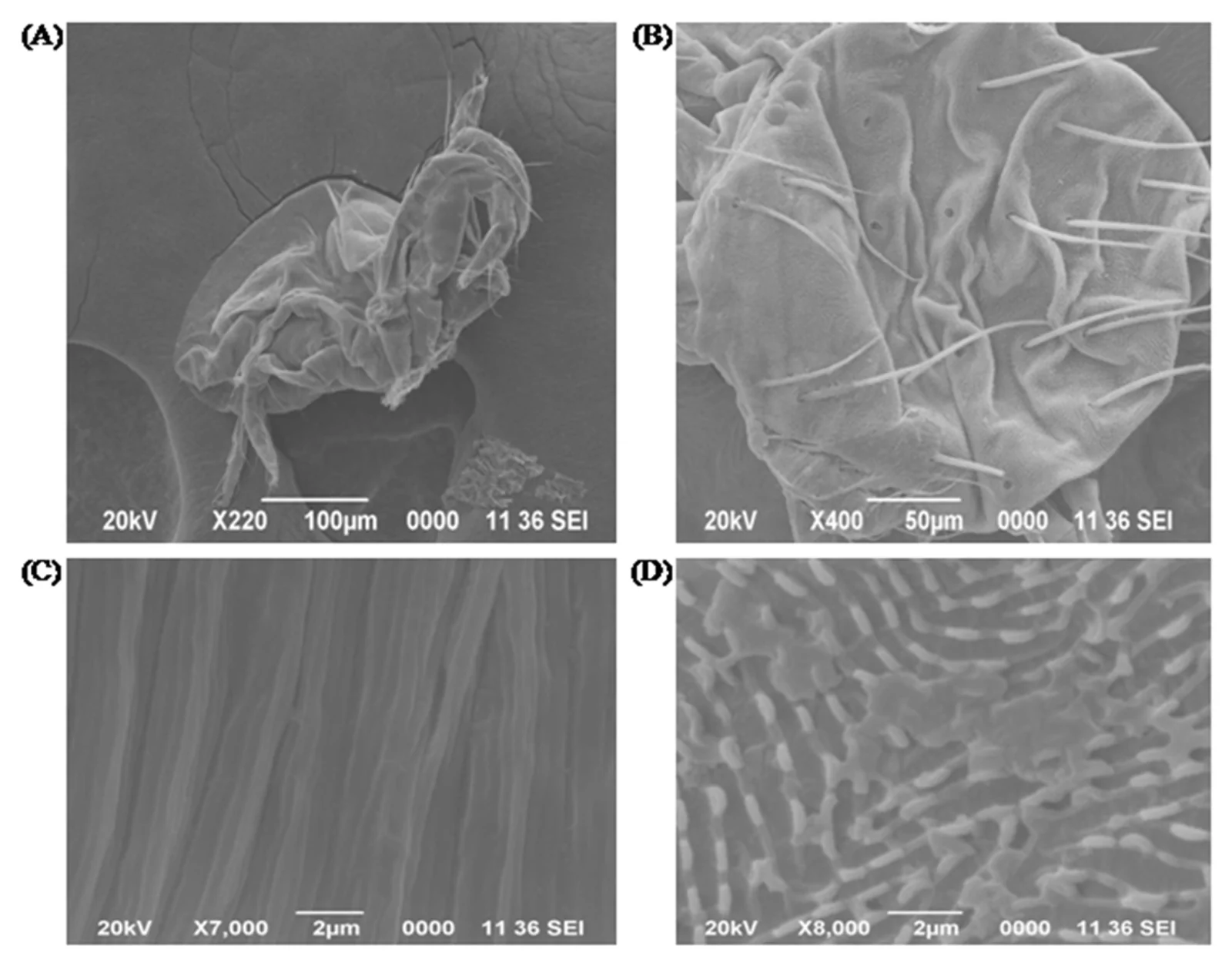
Scanning Electron Microscopy (SEM) images of *O. coffeae* with and without SA-PHSC 0.25% treatment. Section (**A**) shows the entire body of an adult mite before treatment; (**C**) shows the abdominal surface of an adult with control treatment at 20 kV magnification; (**B**) dorsal side of the body of an adult mite after SA-PHSC treatment at 20 kV magnification showing crinkling and constrictions of outer skin; (**D**) granular beads with water-soaked melting like appearance of abdominal skin surface of an adult mite in post-treatment of SA-PHSC at 20 kV magnification.

The relative toxicities of tested insecticides alone and in combination with SA-PHSC (0.25%) in the larval population of *H. talaca* are shown in Figure 1. All the SA-PHSC-mixed insecticidal treatments registered significantly higher percent mortality of *H. talaca* larvae compared to the respective single insecticide treatments in 24 (*F*_6,14_ = 13.20, *p* = 0.0013) and 48 h (*F*_6,14_ = 19.58, *p* = 0.0026) bioassays. However, SA-PHSC mixed with emamectin benzoate was found superior amongst the tested combinations with 96.67 ± 3.34% and 100.0% mortality in both the bioassay intervals (Figure 1a,b). Similarly, in the case of *O. coffeae*, both the tested acaricides in combination with SA-PHSC showed significantly higher (100.0%) mortality of adults, whereas, only 40.00 ± 5.78% to 53.00 ± 3.34% and 77.00 ± 3.33% to 80.00 ± 5.78% mortality were observed in single applications of etoxazole and fenpyroximate, respectively, in 24 (Figure 1c) (*F*_4,15_ = 21.76, *p* < 0.0001) and 48 h (Figure 1d) (*F*_4,15_ = 10.25, *p* < 0.0001) bioassays. Additionally, SEM images showed abnormal cuticular morphology of *H. talaca* larvae (Figure 2) and *O. coffeae* adults (Figure 3) with decay, small micropores, and rupture of the cuticular surface following the application of SA-PHSC.

### 3.2. Field Efficacy of Different Treatments Against H. talaca

Before the application of different treatments, no significant difference of *H. talaca* larval populations was observed, while all the tested combinations with SA-PHSC were found to be more effective than the single insecticides and significantly reduced the larvae after 3 days (*F*_6,14_ = 15.81, *p* = 0.0045) of spray at Soraipani Tea Estate (Table 2). The SA-PHSC in combination with emamectin benzoate showed the significantly highest (97.63%) percent reduction in the larval population at 7 days post application (*F*_6,14_ = 9.79, *p* = 0.0017), however, quinalphos- (94.92%) and deltamethrin-SA-PHSC (95.01%) combinations were statistically at par to each other. Likewise, at Bengdubi Tea Estate, a significantly higher percent reduction in *H. talaca* larval populations was observed in emamectin benzoate combined with SA-PHSC treatment (88.77%), followed by deltamethrin- (86.40%) and quinalphos-SA-PHSC (83.37%) combinations after 3 days of spray (*F*_6,14_ = 18.26, *p* < 0.0001). However, all the tested combinations registered >90% reduction in *H. talaca* larvae at 7 days post-application (*F*_6,14_ = 11.69, *p* = 0.0032) and were significantly higher than the single insecticidal treatments and control.

### 3.3. Field Efficacy of Different Treatments Against O. coffeae

With respect to the field efficacy of different treatments against *O. coffeae*, 7 days post-treatment (*F*_4,15_ = 14.06, *p* = 0.021) at Soraipani Tea Estate, etoxazole and fenpyroximate in combination with SA-PHSC were equally effective in reducing the mean percentage of motile individuals and were significantly different from single acaricides and control treatments (Table 3). Similarly, at Bengdubi Tea Estate, although fenpyroximate-SA-PHSC combination (89.06%) was significantly superior to etoxazole-SA-PHSC (75.87%) treatment in mean percent reduction in *O. coffeae* populations after 7 days of spray (*F*_4,15_ = 24.29, *p* < 0.0001), they were statistically at par with each other at 10 days post-application (*F*_4,15_ = 15.44, *p* = 0.0067) and were significantly higher than single acaricidal treatments.

### 3.4. Yield of Green Tea Leaves

**Figure 4:**
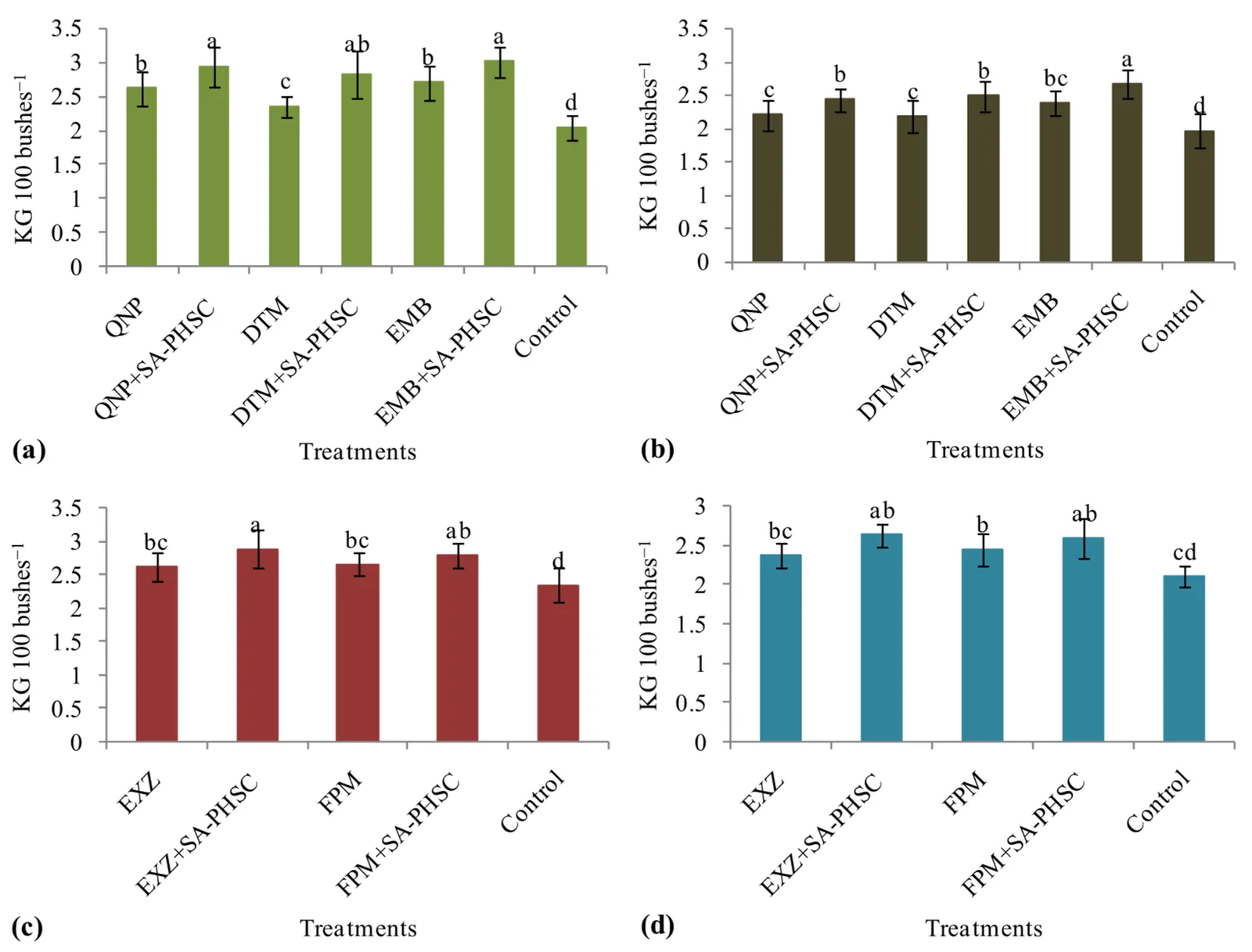
Yield of green tea leaves in different treatments used against *H. talaca* (three replications) at (**a**) Soraipani Tea Estate; (**b**) Bengdubi Tea Estate, and *O. coffeae* (four replications) at (**c**) Soraipani Tea Estate; (**d**) Bengdubi Tea Estate during 2024 and 2025 (pooled). Data are shown as mean ± SE. Different letters in a single parameter indicate significant differences (*p* < 0.05). Refer to Table 1 for the details of treatments.

**Figure 5:**
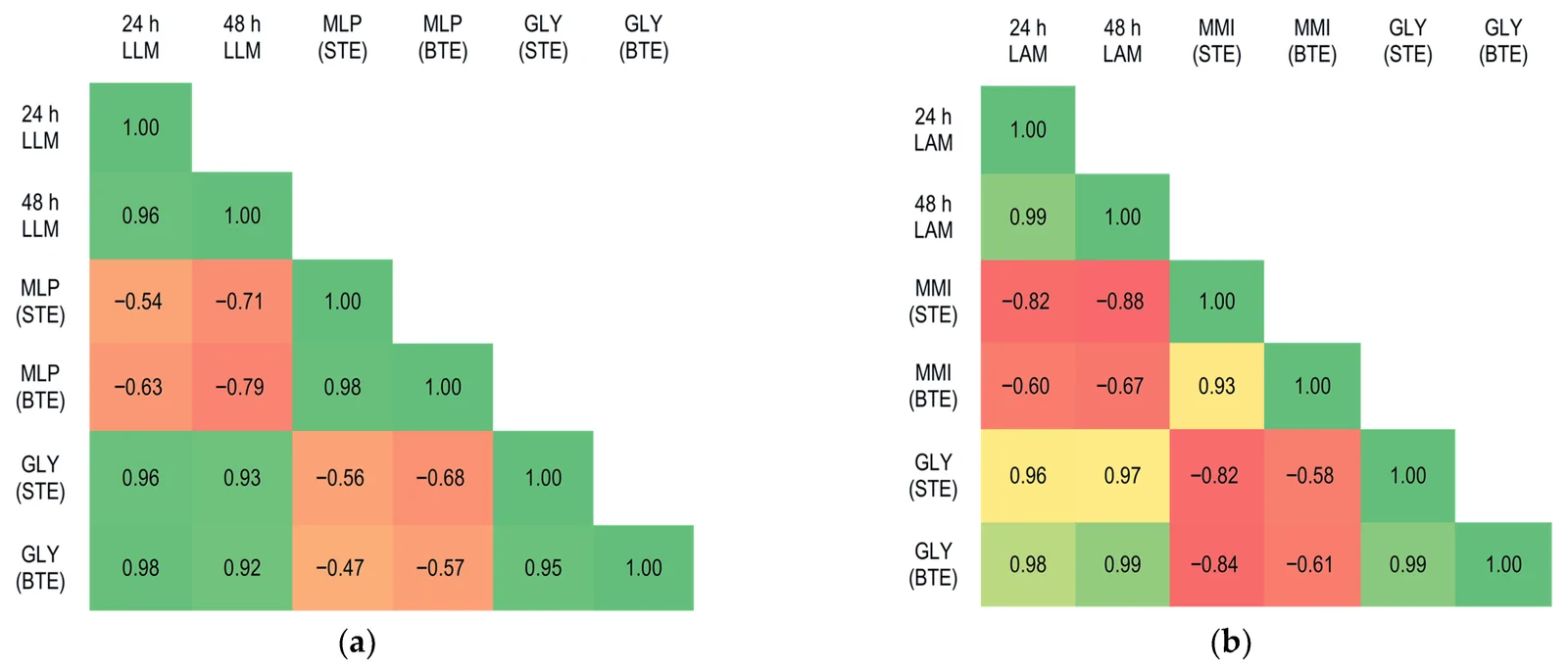
Correlation heat map among different bio-efficacy parameters of the selected treatments on (**a**) *H. talaca* and (**b**) *O. coffeae* in tea. LLM: Laboratory larval mortality; MLP: Mean larval population of *H. talaca*; GLY: Green leaf yield; LAM: Laboratory adult mortality; MMI: Mean motile individuals of *O. coffeae*; STE: Soraipani Tea Estate; BTE: Bengdubi Tea Estate.

The mean yield of green tea leaves in different insecticides-SA-PHSC combination treatments against *H. talaca* was significantly higher compared to the respective single-molecule-treated plots at both Soraipani and Bengdubi Tea Estate (Figure 4a,b). At the former location, emamectin benzoate-SA-PHSC and quinalphos-SA-PHSC combination treatments provided significantly higher yields, followed by the deltamethrin-SA-PHSC combination (*F*_6,14_ = 9.71, *p* = 0.0004); however, quinalphos and deltamethrin in combination with SA-PHSC were statistically at par with each other at the later location (*F*_6,14_ = 17.25, *p* = 0.0009). Similarly, for the tested acaricides-SA-PHSC combination treatments against *O. coffeae*, etoxazole-SA-PHSC combination was found to be significantly superior to fenpyroximate-SA-PHSC combination at Soraipani Tea Estate (Figure 4c) (*F*_4,15_ = 10.23, *p* < 0.0001); however, both the combination treatments were statistically at par with each other and were significantly higher than the respective single acaricide treatments in the yield of green tea leaves at Bengdubi Tea Estate (Figure 4d) (*F*_4,15_ = 12.41, *p* = 0.0006). Additionally, correlation studies indicate that pest mortality and mean pest population in the field showed positive and negative correlations, respectively, with the mean yield of green tea leaves for both *H. talaca* (Figure 5a) and *O. coffeae* (Figure 5b).

### 3.5. Phytotoxicity and Taint Effect

Up to 63 days after SA-PHSC was sprayed on the tea bushes at 1%, 2%, and 4% concentrations, no phytotoxic symptom was observed in the tea leaves. Additionally, post-treatment produced tea samples had an organoleptic score of 6.6–7.0, indicating good liquor, color quality, and strength, and were taint negative.

## 4. Discussion

Insecticide efficacy is a complex process that includes several factors governed by the host plant, target pest, and the environment. Each factor can be optimized to improve insecticide efficiency. The use of tank-mix adjuvants is a common practice in modern agriculture to improve the effectiveness of different agrochemicals in the field. Adjuvants strongly influence the interactions between the pesticide and the target pest [14]; however, paraffinic hydrocarbon-based adjuvants are widely used with insecticides to enhance performance, improve coverage, aid penetration, and reduce the risk of pest resistance to active ingredients [32]. Although several workers reported the effects of using adjuvants with chemical insecticides in the plant systems, scanty information and datasets are available on the relative performance of the existing insecticides with and without adjuvants against arthropod pests and their potential mechanisms in insect systems. Therefore, this study carries a great impact on the increased performance of the existing molecules for the effective management of insect and mite pests of tea plantations, a commercial production system with a limited and restricted pesticide usage.

In the present study, laboratory bioassay results indicate that SA-PHSC increased 41.38%, 36.67%, and 13.33% mortality of *H. talaca* larvae for quinalphos, deltamethrin and emamectin benzoate, respectively. These results might be attributable to the enhanced cuticular penetration of the contact insecticides quinalphos and deltamethrin to the larval cuticle when applied in combination with SA-PHSC [33]. Similar mechanisms may also lead to higher mortality of *O. coffeae* in laboratory bioassays of fenpyroximate and etoxazole mixed with SA-PHSC compared to their single application. Previous studies indicated that the use of tank-mix adjuvants significantly improved the efficacy of organophosphate and synthetic pyrethroid insecticides and showed synergistic interactions against lepidopteran caterpillars of *Spodoptera litura* [34,35]. Additionally, SEM images of *H. talaca* larvae demonstrated clear morphological deformities with numerous open micropores and ruptures on the cuticular surface and validated the hypothesis behind the present results. Hernandez et al. [36] and Rahman et al. [37] also investigated several mechanisms of increased insecticidal toxicity using tank-mix adjuvants against insect pests and corroborate the current investigation. Similarly, SEM images of curling and crinkling of the skin surface of *O. coffeae* adults following the exposure of SA-PHSC alluded to the higher absorption of tested acaricides into the mite body when applied in combination with the tank-mix adjuvant compound, leading to higher mortality. The observed micropores and ruptures in SEM images likely facilitated enhanced penetration of insecticides and acaricides explaining the higher mortality rates. The effectiveness of fenpyroximate in combination with hydrocarbon oil-based adjuvants against *Varroa* mite was investigated by Shannon et al. [38], and they reported the quick knockdown effect on the mite populations due to higher penetration of acaricide through the cuticle with the abnormal biochemical composition of the mite skin surface. Therefore, the potential reason behind the enhanced toxicity of the insecticides and acaricides in combination with SA-PHSC might be the higher cuticular penetration of the active compound through the insect and mite cuticles, followed by the death of the individuals.

In our two-season field experiments, it was observed that the tested insecticides in combination with SA-PHSC registered a higher mean percent reduction in *H. talaca* and field persistency up to 7 days of application. Similarly, the acaricides-SA-PHSC mixtures were found to be superior in the field conditions and significantly reduced the *O. coffeae* populations to a higher extent at 10 days post-application. The enhanced efficacy under the long-term field exposure of the tested insecticides and acaricides mixed with SA-PHSC might be due to the ability of the paraffinic hydrocarbon-based adjuvant to form a ‘depot effect’ by resisting environmental degradation and augmenting active ingredient penetration into the target pests [39]. Holka and Kowalska [40] reported the likelihood of successful pest control using adjuvants with mycoinsecticides with a long-lasting field-effectiveness by increasing the target specificity and counteracting abiotic factors. The results of the present study were also in line with Melo et al. [16] and Hu et al. [12], who substantiated that tank-mix adjuvants have a great impact on the enhancement of insecticide field efficacy by improving the physicochemical properties of spray solutions on the host plant and the target pest. Thus, the SA-PHSC can act as a potent surfactant that optimize the physical properties of the spray solution and thereby reduce pesticide load, environmental contamination, and resistance development by increased efficacy and penetration, reduced spray drift, improved rain fastness, anti-evaporation action, optimized coverage in the plant system and overcoming resistance mechanisms in arthropods. However, several workers documented a negligible or no role of adjuvants in the enhancement of the residual effects of insecticides under the field conditions possibly due to the varied source or nature of the adjuvants, inadequate interaction between adjuvant and active ingredient, different exposure intervals, and the pest intensity [18,41]. Interestingly, the present data show a considerable variation in the mean percent reductions in both *H. talaca* and *O. coffeae* between two different tea plantations which were also reflected in the yield of green tea leaves. This phenomenon might be attributable to the differential field performance of SA-PHSC in combination with tested insecticides or acaricides under varied agroclimatic conditions with some diverse meteorological factors [42,43]. The Bengdubi Tea Estate is located in the Dooars region of the Eastern Himalayan foothills, characterized by the mean lower atmospheric temperature with high moisture and relative humidity, distinct from the Soraipani Tea Estate at the Brahmaputra valley of Assam. It can be hypothesized that the lower temperature and high humidity increased the viscosity of the paraffinic oil of SA-PHSC, which may hinder the droplet formation and coverage and lead to relatively lower pest mortality in the former than in the later region [44]. Lentola et al. [32] highlighted the role of oil-based adjuvants on the efficacy of insecticides in apple plantations under varied weather conditions and fasten the present investigation. Therefore, the SA-PHSC might have a positive impact on the field performance of the recommended insecticides and acaricides against the tested insect and mite pests of tea plantations with a significant influence on the yield of green harvestable tea leaves.

## 5. Conclusions

In conclusion, the SA-PHSC, a paraffinic hydrocarbon-based tank-mix adjuvant, plays a crucial role in increasing the efficacy of quinalphos, deltamethrin, and emamectin benzoate against *H. talaca*, and of etoxazole and fenpyroxymate against *O. coffeae* infesting tea. The SEM images showed abnormal cuticular morphology of *H. talaca* larvae and *O. coffeae* adults following the exposure of SA-PHSC, indicating the potential mechanism and synergistic interaction of this adjuvant in combination with different insecticides and acaricides through enhanced cuticular penetration of the active ingredient followed by higher contact toxicity. Abiotic environmental factors might have a role in the field efficacy of SA-PHSC; however, all the tested insecticides and acaricides in combination with SA-PHSC showed higher efficacy compared to their single application in the field in terms of pest mortality and yield of harvestable tea leaves. Therefore, the use of SA-PHSC as a tank-mix adjuvant in combination with the recommended insecticides and acaricides can be a potential viable option for the tea planters for enhancing field efficacy and restoring susceptibility of tea black looper and red spider mite to the existing molecules for their sustainable and long-lasting management. Nevertheless, the sole insecticidal property of the SA-PHSC, along with its effects on the physiological and developmental traits of *H. talaca* and *O. coffeae*, can be explored in search of some alternative insect and mite pest management strategy in tea.

## Figures and Tables

**Table 1:** Treatment details for laboratory and field bioassay against *H. talaca* and *O. coffeae* in tea.

***Hyposidra talaca***			**Dose (mL or g L^−1^ Water)**	
**Sl. No.**	**Treatment code**	**Treatment details**	**Laboratory**	**Field**
1.	QNP	Quinalphos 25% EC	1.90	1.90
2.	QNP+SA-PHSC	Quinalphos 25% EC + SA-PHSC	1.90 + 2.5	1.90 + 5.0
3.	DTM	Deltamethrin 2.8% EC	0.25	0.25
4.	DTM+SA-PHSC	Deltamethrin 2.8% EC + SA-PHSC	0.25 + 2.5	0.25 + 5.0
5.	EMB	Emamectin benzoate 5% SG	0.50	0.50
6.	EMB+SA-PHSC	Emamectin benzoate 5% SG + SA-PHSC	0.50 + 2.5	0.50 + 5.0
7.	Control	Tap water (pH 7.0)	-	-
***Oligonychus coffeae***			**Dose (mL or g L^−1^ water)**	
**Sl. No.**	**Treatment code**	**Treatment details**	**Laboratory**	**Field**
1.	EXZ	Etoxazole 10% SC	1.00	1.00
2.	EXZ+SA-PHSC	Etoxazole 10% SC + SA-PHSC	1.00 + 2.5	1.00 + 5.0
3.	FPM	Fenpyroximate 5% EC	0.75	0.75
4.	FPM+SA-PHSC	Fenpyroximate 5% EC + SA-PHSC	0.75 + 2.5	0.75 + 5.0
5.	Control	Tap water (pH 7.0)	-	-

For the laboratory and field experiments, each treatment was replicated three and four times for *H. talaca* and *O. coffeae*, respectively. The concentration of SA-PHSC was standardized and double dose (0.50%) was used in the field compared to lab settings (0.25%) to counteract environmental variables that reduce insecticide efficacy.

**Table 2:** Field efficacy of different insecticides alone and in combination with SA-PHSC (0.50%) against larval populations of *H. talaca* (pooled data of 2024 and 2025).

Treatments	Soraipani Tea Estate			Bengdubi Tea Estate		
	PTC	% Reduction (Mean ± SE)		PTC	% Reduction (Mean ± SE)	
		3 d	7 d		3 d	7 d
QNP	89.54	59.49 ± 1.02 ^d^	87.56 ± 0.86 ^c^	105.49	77.99 ± 0.62 ^bc^	83.34 ± 1.14 ^d^
QNP+SA-PHSC	94.83	70.47 ± 0.78 ^c^	94.92 ± 0.97 ^ab^	109.23	83.37 ± 1.50 ^b^	90.16 ± 0.83 ^b^
DTM	92.06	55.20 ± 2.25 ^e^	81.44 ± 1.28 ^d^	98.45	68.85 ± 0.77 ^c^	88.36 ± 2.01 ^c^
DTM+SA-PHSC	88.52	71.45 ± 1.50 ^c^	95.01 ± 0.64 ^ab^	110.53	86.40 ± 1.38 ^ab^	90.04 ± 3.45 ^b^
EMB	97.17	81.93 ± 1.38 ^b^	90.56 ± 0.73 ^b^	99.32	75.78 ± 2.25 ^c^	90.56 ± 1.96 ^b^
EMB+SA-PHSC	94.22	96.51 ± 0.82 ^a^	97.63 ± 0.79 ^a^	95.75	88.77 ± 0.89 ^a^	95.53 ± 1.20 ^a^
Control	90.38	1.97 ± 1.02 ^f^	0.81 ± 1.80 ^e^	108.62	1.96 ± 0.33 ^d^	4.21 ± 1.29 ^e^

PTC: Pre-treatment count (larval population 10 bushes^−1^). d: Days after treatment. Mean values followed by different letters in the column indicate significantly different by DMRT (*p* < 0.05).

**Table 3:** Field efficacy of different acaricides alone and in combination with SA-PHSC (0.50%) against motile stages of *O. coffeae* (pooled data of 2024 and 2025).

Treatments	Soraipani Tea Estate			Bengdubi Tea Estate		
	PTC	% Reduction (Mean ± SE)		PTC	% Reduction (Mean ± SE)	
		7 d	10 d		7 d	10 d
EXZ	7.26	55.48 ± 1.41 ^c^	96.43 ± 0.36 ^a^	7.69	57.80 ± 5.69 ^b^	78.29 ± 2.08 ^b^
EXZ+SA-PHSC	8.49	91.90 ± 1.24 ^a^	96.59 ± 0.42 ^a^	7.82	75.87 ± 6.88 ^b^	89.62 ± 2.17 ^a^
FPM	7.25	68.96 ± 0.32 ^b^	92.96 ± 0.56 ^a^	6.94	37.44 ± 5.86 ^c^	57.82 ± 1.60 ^c^
FPM+SA-PHSC	7.83	93.19 ± 1.23 ^a^	96.43 ± 0.36 ^a^	7.24	89.06 ± 1.91 ^a^	93.93 ± 1.43 ^a^
Control	9.02	0.75 ± 2.05 ^d^	−0.25 ± 0.74 ^b^	7.91	−0.88 ± 0.83 ^d^	−0.97 ± 0.87 ^d^

PTC: Pre-treatment count (no. of motile individuals sq·cm^−1^ leaf surface). d: Days after treatment. Mean values followed by different letters in the column indicate significantly different by DMRT (*p* < 0.05).

## Data Availability

The original contributions presented in this study are included in the article/Supplementary Materials. Further inquiries can be directed to the corresponding authors.

## Supplementary Materials

The following supporting information can be downloaded at: https://www.mdpi.com/article/10.3390/insects17090882/s1, File S1: Refining and production of paraffinic hydrocarbon.
