# Supplementary material for: Unlocking New Potential in Tea Pest Management Using Novel Paraffinic Hydrocarbon-Based Tank-Mix Adjuvant by Boosting Insecticide and Acaricide Efficacy
Source: Insects. 2026 Aug 24;17(9):882. doi: 10.3390/insects17090882 (PMC13607575; doi:10.3390/insects17090882)
Supplement: Supplementary file 1 [file insects-17-00882-s001.zip › insects-4150780-supplementary.pdf]

1. Refining and production of paraffinic hydrocarbons

The hydrocarbon backbone of the SA-PHSC originates from vacuum gas oil (VGO) fractions obtained during atmospheric and vacuum distillation of desalted crude oil. The refining pathway incorporates sequential hydro-processing steps under tightly controlled conditions as follows:

1.1. Hydrocracking and hydroisomerization

The VGO feedstocks are subjected to high-pressure hydrocracking followed by catalytic hydroisomerization over advanced ZSM-22 zeolite and noble metal bifunctional catalysts. This process converts straight-chain paraffins into iso-paraffins, thereby lowering pour points, improving cold-flow properties, and enhancing oxidative stability.

1.2. Severe hydrofinishing

The partially isomerized stream undergoes deep hydrofinishing under hydrogen-rich conditions using Ni-Mo/Co-Mo sulfide catalysts on γ-alumina supports. This stage eliminates trace heteroatoms (S, N, O), saturates residual aromatic moieties, and stabilizes free radicals to produce an ultra-refined paraffinic matrix with negligible saturation.

1.3. Vacuum fractionation

The treated oil is further fractionated in a high-efficiency vacuum distillation column equipped with structured packings, side strippers, and reflux optimization to yield narrow-cut lube oil base stock (LOBS) fractions with tailored viscosity indices and boiling point distributions. After refining, the resulting paraffinic hydrocarbons conform with the specifications as (i) Polycyclic Aromatic Hydrocarbons (PAHs) < 30 ppb, (ii) Total Aromatics < 0.1% (by ASTM D2007), (iii) Saturates > 99.9% (IP 391), (iv) Unsulfonated Residue (USR) 100% (ASTM D483), (v) UV Absorbance (275 nm) < 0.1 absorbance units, (vi) Paraffinic Carbon Content > 70% (13C-NMR quantification), and (vii) Kinematic Viscosity (40 °C) 10–22 cSt.

2. Surfactant integration and adjuvant mechanism

To further impart biological functionality, the paraffinic hydrocarbon fraction is blended with a proprietary non-ionic ethoxylated surfactant system synthesized via controlled ethoxylation of fatty alcohol precursors. This amphiphilic architecture confers:

- Surface Tension Reduction: Facilitates wetting of hydrophobic tea leaf cuticles (dynamic surface tension: < 30 mN/m).
- Spreading Efficiency: Enhances uniform droplet dispersion and minimizes spray beading, quantified through contact angle reduction (< 30° on *Camellia sinensis* leaf lamina).
- Cuticular penetration: Promotes trans-cuticular diffusion of active ingredients by fluidizing the epicuticular wax barrier.
- Retention and Rain fastness: Forms a semi-occlusive film on leaf surfaces, slowing volatilization and wash-off under monsoonal conditions.
- Compatibility: Chemically inert towards organosynthetic insecticides and acaricides, ensuring no antagonistic degradation.
